# Pathophysiology and Treatment of Psoriasis: From Clinical Practice to Basic Research

**DOI:** 10.3390/pharmaceutics17010056

**Published:** 2025-01-03

**Authors:** Yujie Gao, Tianqi Xu, Yu Wang, Yanjinhui Hu, Shaoping Yin, Zhiguo Qin, Hua Yu

**Affiliations:** 1 State Key Laboratory of Quality Research in Chinese Medicine, Institute of Chinese Medical Sciences, University of Macau, Macau 999078, China; mc46217@um.edu.mo; 2 Department of Pharmacy, The First Affiliated Hospital with Nanjing Medical University, Nanjing 210029, China; 047222355@njucm.edu.cn; 3 School of Pharmacy, Jiangsu Provincial Engineering Research Center of Traditional Chinese Medicine External Medication Development and Application, Nanjing University of Chinese Medicine, Nanjing 210023, China; 047421227@njucm.edu.cn (Y.W.); 042121233@njucm.edu.cn (Y.H.); spnzy@njucm.edu.cn (S.Y.)

**Keywords:** psoriasis, pathogenesis, diagnosis, clinical treatment, transdermal drug delivery system

## Abstract

Psoriasis, a chronic inflammatory dermatosis, represents a significant clinical challenge due to its complex pathogenesis and the limitations of existing therapeutic strategies. Current psoriasis diagnoses are primarily clinician-dependent, with instrumental diagnostics serving as adjuncts. Ongoing research is progressively deciphering its molecular underpinnings; the future of psoriasis diagnostics may involve genetic and immunological profiling to pinpoint biomarkers, enabling more accurate and timely interventions. The administration of psoriasis medications, whether oral, injectable, or topical, is associated with a range of side effects and compliance issues. Topical medications, despite their advantages in patient compliance and reduced systemic side effects, are hindered by the altered skin barrier in psoriasis, which impedes effective drug penetration and retention. In recent years, the development of novel transdermal drug delivery systems represents a promising frontier in psoriasis management. Nanotechnology-, microneedle- and dressing-based systems have demonstrated the potential for improved skin penetration, enhanced bioavailability, or extended retention time. Here, we will focus on the latest insights into the etiology, diagnostic methodologies, and therapeutic approaches for psoriasis, with a particular emphasis on the evolution and challenges of novel transdermal drug delivery systems.

## 1. Introduction

Psoriasis is a chronic, recurrent, immune-mediated inflammatory skin disease affecting millions of people worldwide and imposing an estimated annual economic burden of USD 135 billion in the United States [1]. In 2019, the global psoriasis burden included an incidence of 4,622,594, a prevalence of 40,805,386, and a DALY (disability-adjusted life year) of 3,505,736 cases. The incidence rate per 10,000 population is projected to potentially increase by 2030 [2]. Although not studied in every country worldwide, the reported prevalence of this condition varies significantly, ranging from 0.14% in East Asia to 1.99% in Australasia [3]. The global incidence of psoriasis has shown a consistent upward trend over recent decades. Available data indicate that the incidence rate of new cases increased from 92 per 100,000 individuals in 1990 to 99 per 100,000 in 2017 [4]. It can manifest at any age, though it commonly appears in young adults and significantly impacts patients’ quality of life due to its visible and often painful symptoms, including red, scaly plaques that can cause severe itching and discomfort [5]. Beyond the skin manifestations, psoriasis is associated with a range of comorbidities, such as psoriatic arthritis [6], depression, cardiovascular disease, and metabolic syndrome, contributing to both physical and psychological burdens [7] on patients and increasing healthcare costs worldwide.

Current treatment options for psoriasis include topical therapies, phototherapy, and systemic treatments [8]. Some commercially available preparations include fluticasone propionate cream, coal tar lotion, oral methotrexate, etc. [9,10,11]. While these therapies offer symptom relief, none provide a complete cure, and each has limitations [12]. Conventional topical treatments, often considered first-line therapy for mild psoriasis, face challenges in drug penetration [13], resulting in limited efficacy. Additionally, systemic treatments, although effective for moderate to severe cases, come with significant side effects, including hepatotoxicity [14] and nephrotoxicity [15], which limits long-term use. Biologic therapies targeting specific immune pathways have advanced psoriasis management, but their high cost and the potential for immunosuppression-related risks constrain their accessibility [16], especially in low-resource settings. In September 2022, spesolimab was approved for the first time in the US as the first and only biologic targeting IL-36, a key pathway in the disease, to receive breakthrough therapy designation for inclusion in Priority Approval in China, the United States, and other countries and regions (https://www.nhsa.gov.cn/, accessed on 28 December 2024). It significantly prevented episodes of Generalized Pustular Psoriasis for up to 48 weeks [17]. On top of that, on 9 September 2022, Bristol–Myers Squibb’s Deucravacitinib (oral) was approved in the US, ushering in a new era of targeted TYK2 treatment for psoriasis. The unique mechanism of action that the drug possesses is expected to provide a promising oral option to help patients effectively manage their psoriasis [18].

To address these limitations, recent advancements have focused on nano-drug delivery systems as a promising approach for psoriasis treatment. Nanoformulations, including lipid-based and polymer-based nanoparticles, aim to enhance drug delivery, improve skin penetration, reduce dosing frequency, and minimize side effects [19]. These nano-enabled delivery systems provide better bioavailability, controlled release, and targeted delivery, potentially overcoming the barriers posed by conventional therapies [20]. Lee [21] et al. prepared nanoparticles formed by the chemical coupling of the hydrophobic portion of hyaluronic acid with hydrophobic lithocholic acid, which could accumulate and target pro-inflammatory macrophages in the inflamed dermis after transdermal administration, thereby ameliorating the epidermal proliferation and pro-inflammatory response in a mouse model of psoriasis-like skin dermatitis induced by imiquimod and interleukin 23. It also normalizes levels of antimicrobial peptides, tight junction-associated proteins, and major stratum corneum lipid ceramides, thereby restoring skin barrier function against acute psoriasis. Sun [22] et al. reported the use of mung bean-derived nanoparticles to modulate the skin’s immune system to ameliorate psoriasis-like skin inflammation, which exhibited high antioxidant activity, reduced reactive oxygen species, and modulated the immune microenvironment. In imiquimod-stimulated psoriasiform skin, topical application of the nanoparticles alleviated skin inflammation by achieving homeostasis of polarized macrophages and antagonizing activation of the nuclear factor kappa-B signaling pathway.

This review provides a comprehensive and integrated overview of the pathophysiology of psoriasis, covering its various types, characteristic histological features, and the underlying pathological mechanisms that drive disease progression. It further examines current diagnostic approaches and clinical treatment strategies, emphasizing the emerging role of nanotechnology in optimizing drug delivery systems. This advancement holds significant promise for transforming psoriasis management by enabling more effective, patient-centered therapies that address the complex, chronic nature of the condition.

## 2. Clinical Types of Psoriasis

Psoriasis presents in several distinct clinical types, each characterized by unique lesion morphology, affected body regions, and varying responses to treatment. As the most prevalent form of psoriasis, plaque psoriasis is characterized by round or flat, erythematous plaques with a distinctive pink base covered by silvery-white scales. These plaques commonly occur on areas such as the elbows, knees, scalp, and lower back [23]. The condition is often accompanied by varying degrees of pruritus, which can range from mild to severe, significantly affecting patient comfort and quality of life.

Guttate psoriasis is identified by small, discrete red papules, typically ranging from 1 to 10 mm in diameter, with minimal scaling [24]. Guttate psoriasis often appears suddenly, often triggered by a streptococcal infection, such as strep throat [25].

Pustular psoriasis is further divided into generalized pustular psoriasis (GPP), localized forms such as palmoplantar pustulosis (PPP) and acrodermatitis continua of Hallopeau (ACH) [26]. GPP is marked by a rapid eruption of sterile pustules on a background of red, inflamed skin, often accompanied by systemic symptoms like fever, fatigue, and muscle weakness, which can necessitate urgent medical attention due to potential complications [27]. PPP manifests as sterile pustules on the palms and soles, associated with erythema, scaling, and thickening of the skin (keratoderma) [28]. ACH is a condition characterized by recurrent pustules on the tips of the fingers. Patients may experience osteolysis, which has a significant impact on quality of life [29].

Erythrodermic psoriasis is a rare but severe form of psoriasis, characterized by extensive erythema and desquamation covering more than 75% of the body’s surface area [30]. Erythrodermic psoriasis often arises as an exacerbation of another psoriasis subtype or in response to triggers such as infection, withdrawal of systemic psoriasis treatment, or stress. Due to its widespread inflammation, this form can lead to systemic complications, including fluid and electrolyte imbalances, thermoregulation disturbances, and increased risk of infection [31].

Psoriatic arthritis (PsA) is a chronic inflammatory arthritis associated with psoriasis. PsA commonly involves the small joints of the hands and feet, but larger joints such as the hips, knees, and spine may also be affected. Symptoms include joint pain, swelling, and stiffness, which can be disabling and may occur either in conjunction with or independently of skin lesions [32].

## 3. Pathogenesis of Psoriasis

### 3.1. Histological Features

Psoriatic lesions are characterized by notable epidermal hyperplasia, parakeratosis, and the presence of pathognomonic features such as Munro’s microabscesses [33] and Kogoj spongiform pustules [34]. Additional key histopathological findings include a reduced or absent granular layer, dilated and elongated dermal capillaries, and a significant infiltration of inflammatory cells, particularly T cells, within the dermis and epidermis. These histological changes underlie the clinical manifestations of psoriasis, contributing to hallmark symptoms such as erythema and scaling [35].

### 3.2. Etiology

Psoriasis has a multifactorial etiology, with a significant genetic predisposition contributing to disease risk [36]. To date, over 60 susceptibility loci have been identified, with the HLA-Cw6 allele being particularly associated with early-onset psoriasis [37]. Environmental factors, including psychological stress, physical trauma (known as the Köbner phenomenon) [38], and lifestyle choices, such as smoking, further influence disease expression. Notably, while smoking is known to increase the risk of developing psoriasis in the general population, recent studies indicate that it may, paradoxically, reduce the likelihood of developing psoriatic arthritis among those affected by psoriasis [39].

### 3.3. Cell Signaling Pathways Related to Psoriasis

**Figure 1:**
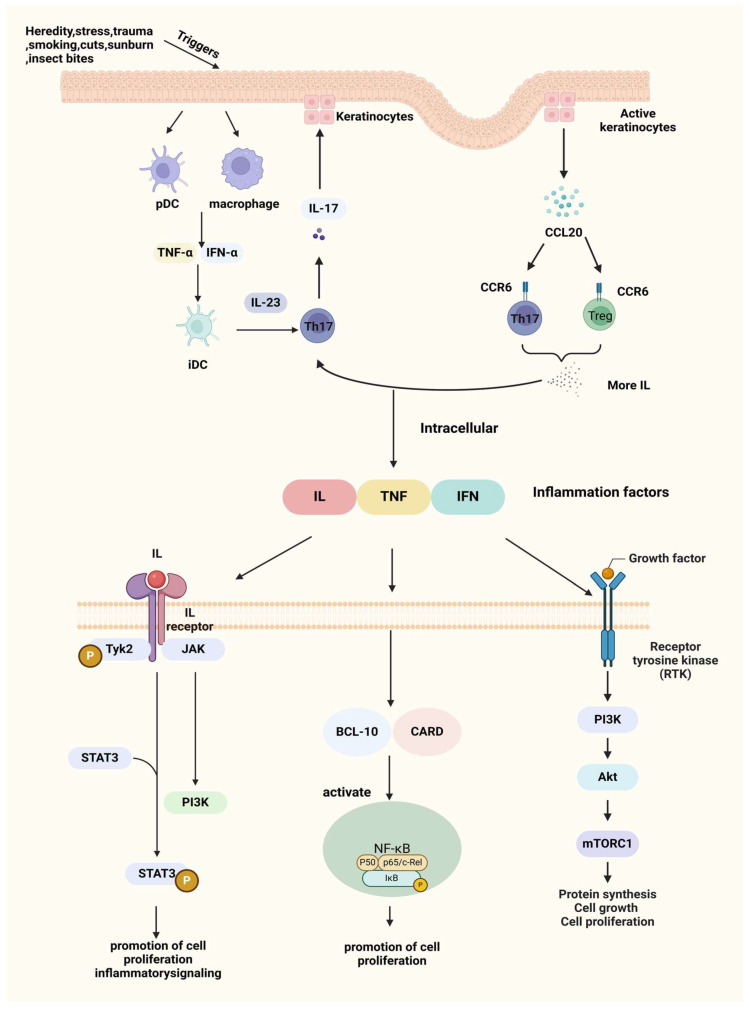
Triggers and pathogenesis of psoriasis. After IL-23/Th17axis is activated, a large number of inflammatory factors are produced, which are further exacerbated by the CCL20-CCR6 pathway, e.g., IL, TNF, IFN, etc., which bind to receptors on the cell membrane and further transduce inflammatory signals inside the cell, exacerbating the symptoms of psoriasis. Created with BioRender.com.

The pathogenesis of psoriasis is intricately linked to T cell immunity. The disease process begins with the activation of antigen-presenting cells (APCs) within the skin, such as dermal dendritic cells and epidermal Langerhans cells, in response to external or internal antigens, including skin trauma or infection. Upon activation, these APCs migrate from the skin to nearby lymph nodes, where they present antigens to naive CD45RA+ T cells, initiating T cell activation [40]. Then, T cells migrate into the affected skin and release cytokines that drive keratinocyte hyperproliferation, disrupt normal cellular differentiation, and perpetuate inflammatory responses through positive feedback loops in cell signaling. This cascade results in the sustained formation of psoriatic skin lesions. Therefore, cell signaling pathways are central to the pathogenesis of psoriasis. Understanding these pathways is crucial for developing effective therapeutic approaches, as they represent key intervention points in managing the disease. Figure 1 is a depiction of the current psoriasis cell signaling pathway.

#### 3.3.1. Interleukin-23/T Helper Cell (IL-23/Th17) Axis

Under pathogenic stimuli, myeloid dendritic cells (DCs) become activated and produce excessive levels of inflammatory cytokines, such as IFN-α, tumor necrosis factor-alpha (TNF-α), and IL-6. These cytokines, in turn, promote the increased secretion of IL-12 and IL-23 [41]. In response to IL-23, Th17 cells produce high levels of IL-17, which acts on keratinocytes, leading to epidermal hyperplasia, activation of innate immune responses, recruitment of leukocytes to the skin, and further production of pro-inflammatory cytokines such as IL-1β, IL-6, and IL-8 [42]. Blocking IL-17 can disrupt this pathological cycle, effectively reducing inflammation and alleviating clinical symptoms of psoriasis [43].

#### 3.3.2. Macrophage Inflammatory Protein 3 Alpha/Chemokine (C-C Motif) Ligand 20-C-C Chemokine Receptor 6(MIP-3α/CCL20-CCR6) Pathway

The CCL20/CCR6 axis has long been associated with inflammation. CCL20 (also known as MIP-3α) acts as a chemotactic factor that, through binding with its receptor CCR6, is crucial for transporting IL-17A-producing T cells from the bloodstream to the skin during the progression of psoriasis [44]. CCL20 is primarily produced by activated keratinocytes, while CCR6 is predominantly expressed on dendritic cells (DCs) and T cells, especially Th17 cells, serving as a marker for Th17 and regulating lymphocyte migration. In psoriatic skin lesions, keratinocyte activation leads to the upregulation of CCL20 expression, enhancing its binding to CCR6. This increased binding attracts more Th17 cells to the site, which produce cytokines like IL-17, further driving the IL-23/Th17 axis and perpetuating the inflammatory response in psoriasis [45].

#### 3.3.3. Nuclear Factor Kappa-B (NF-κB) Signaling Pathway

Additionally, the Caspase Recruitment Domain 14 (CARD14) gene (also known as CARMA2), which is highly expressed in psoriatic skin, has been shown to specifically interact with BCL10, a protein known to positively regulate apoptosis and NF-κB activation. When expressed in cells, CARD14 activates NF-κB and induces the phosphorylation of BCL10 [46], further promoting these signaling pathways.

#### 3.3.4. JAK (Janus Kinase)/STAT (Signal Transducer and Activator of Transcription) Signaling Pathway

The JAK family, including JAK1, JAK2, and TYK2, activates STAT3, driving Th17 cell activation and cytokine production [47]. STAT3 is involved in regulating cell growth and apoptosis and plays an essential role in the differentiation of Th17 cells (a T cell subset that secretes IL-17) [48]. When the skin is exposed to external stimuli, dendritic cells and monocytes in the dermis release IL-23 to induce Th17 cells to produce large amounts of cytokines to activate STAT3 in keratinocytes (KCs), which will drive epidermal hyperproliferation, promote leukocyte recruitment, and facilitate the formation of mature psoriatic plaques [49].

Tyrosine kinase 2 (TYK2), a key regulatory molecule, has emerged as a promising therapeutic target due to its pivotal role in mediating pro-inflammatory and immune-stimulatory signaling driven by cytokines such as IL-12, IL-23/IL-17, and type I interferons [50]. To avoid affecting other JAK family members, researchers are focusing on TYK2’s regulatory domain. By targeting this domain with small-molecule ligands, it is possible to selectively inhibit TYK2 signaling without interfering with other JAK pathways [51].

#### 3.3.5. Phosphatidylinositol 3 Kinase (PI3K)/Protein Kinase B (AKT) Signaling Pathway

The PI3K/AKT axis plays a significant role in inflammatory skin diseases, with a particular focus on its downstream target, the mechanistic target of rapamycin (mTOR), which is central to some of the most common inflammatory skin conditions. Activation of mTOR promotes keratinocyte hyperproliferation and inhibits their differentiation, contributing to the pathological manifestations of psoriasis [52]. The Forkhead box O (FoxO) transcription factors are negatively regulated by AKT-mediated phosphorylation and modulate the expression of numerous genes involved in controlling cell proliferation [53]. Activation of FoxO blocks cell proliferation and induces a quiescent state, making FoxO levels or activity a potential prognostic marker for disease progression in psoriasis patients. Among the FoxO family, FoxO3, which is widely expressed across various human organs and tissues, shows promise as a potential therapeutic target [54].

## 4. Diagnosis and Clinical Treatment of Psoriasis

### 4.1. Diagnosis of Psoriasis

The diagnosis of psoriasis is primarily based on the characteristic clinical manifestations, such as scaly erythematous plaques, along with patient history, including disease onset, progression, associated symptoms, and response to previous treatments [55]. When necessary, histopathological examination can be used to confirm the diagnosis. To enhance diagnostic accuracy, clinicians often utilize auxiliary tools such as dermoscopy and digital radiography. Table 1 shows various auxiliary diagnostic methods for diagnosis of psoriasis. Diagnostic criteria for psoriasis continue to evolve with advancements in clinical research.

### 4.2. Clinical Treatment of Psoriasis

**Figure 2:**
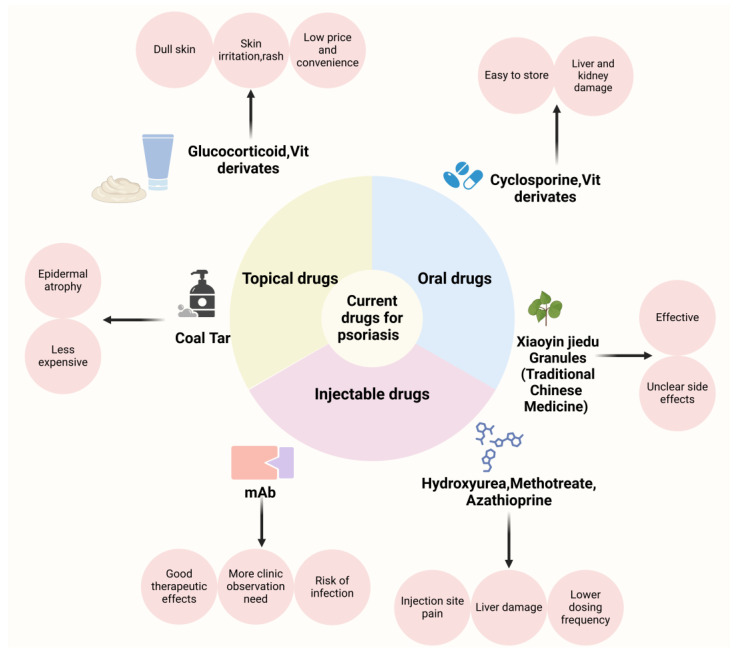
Overview of the types, advantages, and disadvantages of medications for the clinical management of psoriasis. Created with BioRender.com.

The primary goals in the clinical management of psoriasis are to control disease activity, alleviate symptoms, prevent recurrence, and improve the patient’s quality of life. Currently, the Psoriasis Area and Severity Index (PASI) is the standard measure for evaluating the effectiveness of psoriasis treatments. The PASI score considers both the extent of the affected skin area and the severity of lesions, including erythema, thickness (induration), and scaling. Clinical trials for the treatment of psoriasis have traditionally considered the PASI 75 (referring to a 75% improvement in PASI compared to the pre-treatment one) as the ultimate therapeutic index [64]. In addition, with the use of biologics, PASI 100 and PASI 90 are gradually used as clinical benchmarks [65], respectively. Many treatments have been clinically proven effective in alleviating symptoms and pain associated with psoriasis; however, most still present significant limitations. As shown in Figure 2, a range of well-established oral, systemic, and topical therapies are available on the market. Table 2 shows various treatment methods for psoriasis in a clinic.

#### 4.2.1. Oral Medicines for Psoriasis

Conventional oral treatments for psoriasis primarily include immunosuppressants (such as methotrexate and cyclosporine), retinoids, and corticosteroids. Methotrexate is a folate reductase inhibitor that suppresses cell proliferation and reduces inflammation, making it suitable for patients with moderate to severe psoriasis. Several organ toxicities have been characterized during the clinical use of methotrexate, including hepatotoxicity, nephrotoxicity, pulmonary toxicity, and gastrointestinal toxicity. In the clinical setting, renal injury is the main dilemma limiting the utility of methotrexate [97]. Apremilast, a phosphodiesterase-4 inhibitor, regulates cyclic adenosine monophosphate (cAMP) levels, resulting in a broad suppression of proinflammatory mediators and a subsequent reduction in tumor necrosis factor-alpha (TNF-α) activity. Phase III randomized controlled trials, including PALACE and ACTIVE, demonstrated the efficacy of apremilast in improving clinical and patient-reported outcomes in psoriatic arthritis (PsA) patients, regardless of prior disease-modifying anti-rheumatic agent (DMARD) experience [67]. Cyclosporine [98], another immunosuppressant, reduces skin inflammation by inhibiting T cell activation and is appropriate for severe cases of psoriasis. However, long-term use may lead to adverse effects such as liver and kidney dysfunction and bone marrow suppression. Retinoids, such as acitretin [99], regulate cell differentiation and proliferation, and are effective for plaque, pustular, and erythrodermic psoriasis. A double-blind study involving 38 patients with stable, plaque-type psoriasis showed that the most frequent adverse effects of acitretin included cheilitis, peeling of the palms and soles, and alopecia, similar to those observed with etretinate therapy. Laboratory abnormalities primarily involved elevated serum triglycerides, with less frequent increases in serum cholesterol and liver transaminase levels [68]. Glucocorticosteroids [100], such as prednisone, offer potent anti-inflammatory and immunosuppressive effects. Due to potential side effects with long-term use (including osteoporosis, muscle atrophy, skin thinning, mood swings, immune suppression, glaucoma and cataracts, gastrointestinal issues, impaired growth, and increased bleeding risk), corticosteroids are typically recommended for short-term treatment. Zkib [69] et al. presented the case of a 43-year-old woman with multiple sclerosis who developed symptomatic bradycardia following three days of high-dose methylprednisolone treatment.

Additionally, certain traditional Chinese medicine (TCM) formulations are used in psoriasis management, which refers to medicinal substances used for disease prevention and treatment based on TCM principles, including raw herbal materials, processed herbal pieces, and proprietary Chinese medicines. TCM differs from Western medicine in its theoretical foundation, treatment principles, sources of medication, and methods of application [101]. In TCM, psoriasis is often attributed to blood-related issues such as blood heat, blood dryness, and blood stasis and is associated with dysfunction in organs like the liver and kidneys. Formulations, such as Xiaoyin Jiedu Granules [102], and ingredients are as follows: 30 g of Shuiniujiao, 15 g of Shengdihuang, 15 g of Danpi, 15 g of Chishao, 15 g of Baihuasheshecao, 15 g of Quanshen, 20 g of Zicao, 10 g of Shenghuaihua, 15 g of Tufuling, 15 g of Daqingye, and 20 g of Rendongteng. These are thought to cool, detoxify, and nourish the blood and may be used, but treatment is usually tailored to individual conditions. A clinical report with 25 psoriasis vulgaris patients with a blood–heat pattern indicated that the Xiaoyin Jiedu Granules significantly lower the psoriasis area severity index (PASI) and itching scores and improve dermatology life quality index (DLQI) by reducing the number of Th9 and Th17 cells and the levels of their related cytokines [72].

#### 4.2.2. Injectable Medications for Psoriasis

General psoriasis injections use medications such as methotrexate, azathioprine, and hydroxyurea [103]. They are fast acting, give relatively accurate doses, reduce the frequency of administration for patients, and are relatively more affordable. However, there are inevitable side effects associated with the injections, such as redness, swelling, and itching at the injection site. Gonzalez-Moure et al. reported a case of a 53-year-old woman with a 2-month history of recurrent cutaneous lesions appearing a few days after methotrexate (MTX) injections. Dermatological examination revealed several 4–5 cm painful, erosive, erythematous-violaceous plaques with delicate crusting on the surface located on the lower abdomen [73]. Moreover, due to the lack of targeting of these agents, they may damage the normal tissues of the body [104]. Due to the side effects, hydroxyurea (HU) is no longer widely used for the treatment of psoriasis. The primary side effects of HU include relatively common and benign reactions such as hyperpigmentation, xerosis, and skin atrophy. However, more serious cutaneous complications, including leg ulcers and nonmelanoma skin cancers, can occur in a significant number of patients [74]. Azathioprine, a relatively safe immunosuppressant, was used to treat a 54-year-old Caucasian male with ANCA-associated vasculitis presenting with hemoptysis, arthralgia, impaired kidney function, active urine sediment, and a positive p-ANCA titer. Two weeks after starting azathioprine, the patient developed a painful erythematous maculopapular rash and fever. A skin biopsy confirmed classical features of Sweet’s syndrome [75]. Therefore, in order to obtain better therapeutic results, biological agents were developed.

Biologics are a class of targeted therapies used in the treatment of psoriasis, designed to alleviate inflammation and skin symptoms by interfering with specific immune pathways [105]. The main types of biologics used for psoriasis include TNF-α inhibitors, IL-12/23 inhibitors, IL-23 inhibitors, IL-17A inhibitors, and IL-36R inhibitors. Biologics offer high efficacy and favorable safety profiles, providing rapid improvement in skin symptoms and reducing the impact of the disease on patients’ quality of life. Due to their targeted mechanism of action, biologics generally have fewer side effects compared to traditional therapies. However, biologics may increase the risk of infection, and their long-term efficacy and safety require further investigation. Additionally, patients may develop anti-drug antibodies, potentially reducing treatment effectiveness over time. Mohammed et al. reported a patient with Crohn’s disease who developed infliximab-induced psoriasis vulgaris after starting infliximab treatment [78]. Scheinfeld summarized the most common side effects of Adalimumab are injection site reactions. It significantly increases the risk of serious infections, including tuberculosis reactivation, deep fungal infections, and atypical infections, with a two-fold risk of severe infections reported in the Premier trial. Rare side effects include the worsening or onset of congestive heart failure, lupus-like syndrome, lymphoma, significant cytopenias, multiple sclerosis or other neurological disorders, pancytopenia, and elevated transaminases [79]. A 20-week interim analysis of a postmarketing surveillance study was conducted to evaluate the safety and effectiveness of guselkumab in 411 and 245 patients, respectively, who were treated between May 2018 and October 2020 in Japan. Adverse drug reactions (ADRs) were reported in 6.6% of patients, with serious ADRs in 2.2%. The most common ADRs were classified under “Infections and infestations” (2.4%), with nasopharyngitis being the most frequent (0.7%) [81]. Secukinumab, the first IL-17A inhibitor approved for moderate-to-severe psoriasis, is generally well-tolerated with a favorable safety profile. Common adverse effects reported in clinical trials include nasopharyngitis, diarrhea, upper respiratory tract infections, pruritus, and headache. Additional adverse events occurring in >1% of patients include rhinitis, oral herpes, urticaria, and rhinorrhea, while neutropenia was observed in 1% of patients. Mucocutaneous candidiasis was reported at a rate of 3.55 per 100 patient-years with a 300 mg dose. Moreover, it may cause a severe cutaneous reaction [84]. The high cost of biologics can also impose a financial burden on patients [106].

#### 4.2.3. Topical Therapies for Psoriasis

Topical therapy remains an essential treatment approach for psoriasis, especially for patients with mild to moderate disease. These medications can be applied directly to affected skin areas, circumventing the liver’s first-pass effect, offering rapid efficacy, enhancing patient adherence, reducing adverse effects, and enabling controlled, flexible dosing. Common topical agents include emollients, moisturizers, vitamin D3 derivatives, corticosteroids, calcineurin inhibitors, and tar preparations [107]. Combination formulations, such as calcipotriol with betamethasone, clobetasol propionate with retinoids, and tazarotene with betamethasone, have been developed to enhance therapeutic efficacy, reduce adverse effects, and improve patient convenience. However, long-term use of topical medicine may lead to local adverse reactions, such as skin atrophy and capillary dilation. Calcipotriol, a vitamin D analog, binds to the vitamin D receptor in keratinocyte nuclei to inhibit their proliferation. However, its long-term use is associated with local skin reactions, which often compromise patient adherence. Additionally, approximately 20% of patients fail to respond to calcipotriol treatment, highlighting its limited efficacy in certain cases [91]. Coal tar exhibits anti-inflammatory, antimicrobial, antipruritic, and cytostatic properties, making it effective for treating psoriasis. Long-term exposure to coal tar can cause allergic dermatitis, folliculitis, occupational acne, epidermal atrophy, and hyperpigmentation. Skin irritation, phototoxic reactions, and burns are the common adverse effects [92]. Topical corticosteroids, such as fluticasone propionate and mometasone furoate, are widely used in the treatment of psoriasis due to their effectiveness in relieving symptoms. However, their use may lead to adverse effects. Clinical trials involving fluticasone propionate reported only local cutaneous reactions, which affected up to 19% of patients. These included pruritus, burning, dryness, stinging, folliculitis, irritation, or exacerbation of the dermatological condition [93,94,95]. Topical tacrolimus serves as an alternative to corticosteroids by inhibiting cytokine production by blocking the activation of the nuclear factor of activated T cells (NF-AT). Tacrolimus does not impair collagen synthesis, avoiding the risk of skin atrophy. However, its efficacy in treating plaque-type psoriasis is limited, likely due to poor penetration through psoriatic plaques [96]. For patients with widespread lesions, topical application may not be practical [108].

## 5. Challenges and Innovations in Transdermal Drug Delivery System for Psoriasis Treatment

**Figure 3:**
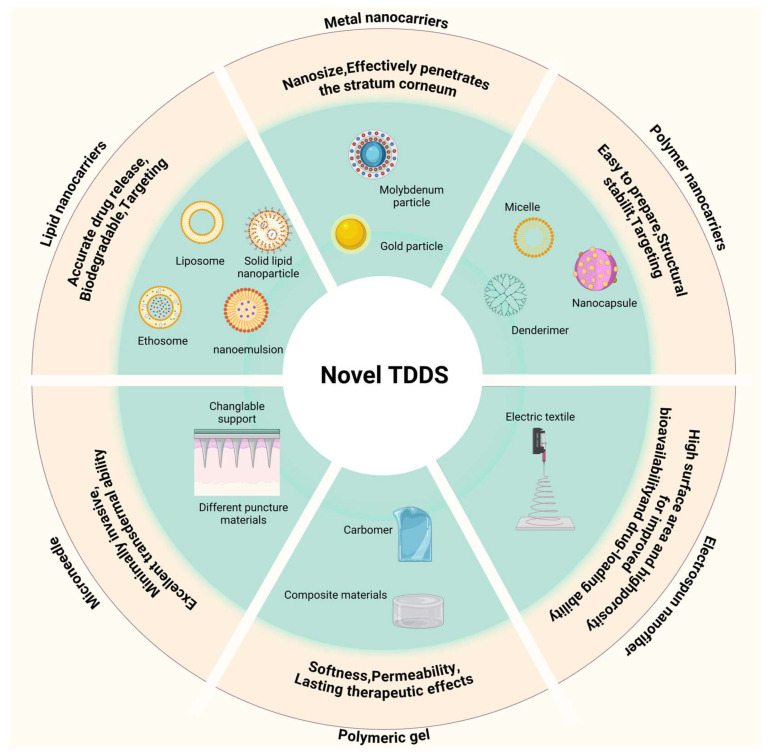
Summary of novel transdermal drug delivery system carriers. Created with BioRender.com.

Transdermal drug delivery systems (TDDSs) are designed to administer therapeutic agents through the skin, enabling controlled, systemic, or localized drug release [109,110]. Transdermal methods allow for localized, controlled drug release, which can enhance therapeutic efficacy and improve patient compliance through ease of application [111]. However, the dense, hyperkeratotic structure of psoriatic lesions significantly impedes drug penetration. Conventional topical formulations, such as ointments and creams, often lack the permeability to pass through the thickened stratum corneum, resulting in limited absorption [112]. Furthermore, due to the reduced moisture and ceramide levels alongside increased cholesterol content of the psoriatic lesions, it is less receptive to drug diffusion [113]. Novel TDDSs can enhance drug penetration efficiency and bioavailability while reducing side effects. In recent years, novel TDDSs, including nanotechnology-based transdermal delivery systems, microneedle-based transdermal delivery systems, and dressing-based transdermal delivery systems, have been widely applied in the treatment of psoriasis. In Figure 3, we briefly schematize these carriers.

### 5.1. Nanotechnology-Based Transdermal Delivery for Psoriasis Treatment

**Figure 4:**
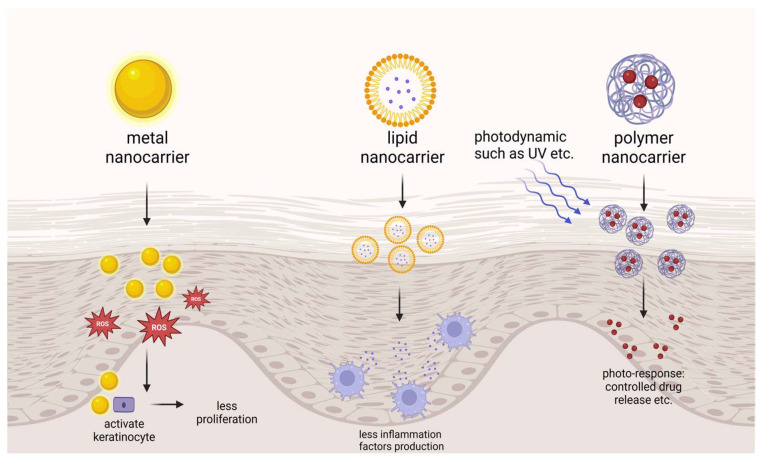
Introduction to nanocarriers in psoriasis treatment. Created with BioRender.com.

Nanotechnology-based drug delivery systems possess unique advantages in the field of dermal/transdermal delivery, particularly for the treatment of psoriasis: they can fluidize the stratum corneum through properties such as shape, size, surface charge, and the balance between hydrophilicity and hydrophobicity; their nanoscale dimensions allow for intimate contact with the skin surface, thereby facilitating the penetration of drugs through the skin, making nanocarriers effective at breaching the skin’s barrier functions [114]; they are capable of maintaining drugs at the skin surface through controlled release mechanisms [115]; they can offer a sealing effect to reduce transepidermal water loss [116]; they can provide targeted action by modifying with cell-specific ligands [117]. In recent years, a variety of nanotechnology-based transdermal delivery, such as metal nanocarriers, polymer nanocarriers, and lipid nanocarriers, have been developed for psoriasis applications and are listed in Table 3. A profile of the nanocarriers is shown in Figure 4.

#### 5.1.1. Metal Nanocarriers for Psoriasis Treatment

Metal nanocarriers like gold nanoparticles (AuNPs) have demonstrated certain advantages in psoriasis treatment that allow them to effectively penetrate the skin’s stratum corneum and act directly on epidermal cells. In psoriatic keratinocytes, high levels of reactive oxygen species (ROS) and inflammatory factors create a vicious cycle of inflammation. To disrupt this cycle, Guo [137] et al. synthesized nanoscale molybdenum particles with an average size of 93.2 nm, which showed no impact on HaCaT cells at concentrations up to 300 ppm and targeted the inflammatory NF-κB pathway by reducing levels of p-p65. In a mouse model of psoriasis induced by imiquimod, scoring and histological staining further indicated the anti-inflammatory effects of this material. Han [138] et al. developed a novel three-component alkyl-terminated gold nanoparticle featuring a 3 nm gold core and a 1000 Da polyethylene glycol (PEG) shell modified with 30% octadecyl chains. This sub-15 nm nanoparticle maintains colloidal stability within the skin due to the octadecyl chain modification, enhancing epidermal cell uptake. By inhibiting genes associated with hyperproliferation and inflammation within the downstream IL-17 signaling pathway, these AuNPs show potential in preventing psoriasis. Optimized alkylation of the Au core improves cellular uptake and provides a more pronounced inhibition of inflammatory genes, underscoring the therapeutic efficacy of these AuNPs in psoriasis treatment.

#### 5.1.2. Polymer Nanocarriers for Psoriasis Treatment

Polymer-based nanocarriers typically range in size from 10 to 1000 nm, with drugs either encapsulated within or bound to the nanocarrier matrix. These carriers are easy to prepare, exhibit targeted delivery capabilities, and offer high safety, making them widely used in the treatment of various diseases. Additionally, their robust matrix structure provides high structural stability when applied topically, allowing the formulation to maintain integrity over extended periods and enhancing drug penetration through the skin. The most commonly used polymer-based nanocarriers for psoriasis treatment include polymeric micelles, nanocapsules, dendrimers, and nanoemulsions. Arun Sontakke [139] et al. Design of fatty acid surfactant-based micellar gels for delivery of Apremilast. This carrier extended the release of Apremilast to up to 36 h, increased local drug concentrations, and reduced systemic exposure, facilitating topical delivery. Wang [140] et al. constructed labeled cell membrane-derived nanovesicles coated with IR-780 nanoparticles (N3-NV-INPs) using N3 to enhance PDT and PTT treatment, but the lack of specific targeting of proteins and cells may cause damage to other cells, and the safety has to be examined. Yu [141] et al. designed photoresponsive dendritic mesoporous silica nanoparticles loaded with Erianin, showing the ability to modulate drug release using UV light as an external stimulus. The release rate of Erianin was significantly optimized and, therefore, will be able to reduce fluctuations in blood drug concentration and side effects of the drug. Mohamed Ashraf [142] et al. attempted to use Eucalyptus oil nanoemulsion for the treatment of psoriasis. The optimized fluticasone propionate (FP) loaded eucalyptus oil nanoemulsion formulation prepared by a low-energy method was a successful approach to optimize a safe formulation without the use of organic solvents, thus enabling maximum incorporation of FP that achieves a combined anti-inflammatory effect with eucalyptus oil for healing of psoriatic plaques. Although the topical administration of Tacrolimus is effective in the treatment of various inflammatory skin diseases, commercially available transdermal delivery formulations of Tacrolimus remain a challenge due to its high hydrophobicity and high molecular weight. Wan [143] et al. developed a Tocopheryl Polyethylene Glycol 1000 Succinate (TPGS) as an adjuvant of nanoemulsions for the treatment of a psoriasis model. The results showed that TPGS nanoemulsion could enhance the solubility and permeability of Tacrolimus and exhibit an adjuvant effect against psoriasis, and thus is a promising nanoscale Tacrolimus system for the effective treatment of psoriasis.

#### 5.1.3. Lipid Nanocarriers for Psoriasis Treatment

Lipid-based carriers, typically composed of physiological lipids, are highly biocompatible and are widely employed in pharmaceutical applications. These nanocarriers provide a spectrum of benefits, such as precise drug release, improved stability, biodegradability, targeted drug delivery, enhanced drug payload, and economic efficiency. Particularly, lipid nanocarriers like nanostructured lipid carriers and solid lipid nanoparticles are capable of carrying both hydrophobic and hydrophilic therapeutic agents. A range of lipid nanocarriers, including liposomes, ethosomes, and solid lipid nanoparticles, have shown significant potential in the effective delivery of anti-psoriatic medications. Cationic liposomes consisting of DC-Chol, cholesterol, and anionic liposomes consisting of lecithin, cholesterol, and tetramyristoyl cardiolipin were developed by Sindhu Doppalapudi [144] et al. for topical delivery of psoralen (PSR); this enhanced the skin permeability of PSR. Teng Guo [145] et al. performed surface modification of glycyrrhetinic acid-D-α-tocopherol acid polyethylene glycol succinate (GA-TPGS) on curcumin-loaded ethanol vesicles to construct curcumin-loaded GA-TPGS-modified multifunctional ethanol vesicles (Cur@GA-TPGS-ES), which exerted synergistic therapeutic effects on psoriasis. Guohua Ren [146] et al. embedded calcipotriol in a solid lipid with Compritol 888 ATO as the oil phase and Poroxam 188 (F188) as the emulsifier, forming a new formulation with strong keratin penetration, slow release, and targeted action, thus improving the topical therapeutic effect of the drug and reducing the occurrence of skin irritation.

### 5.2. Microneedle-Based Transdermal Delivery for Psoriasis Treatment

**Figure 5:**
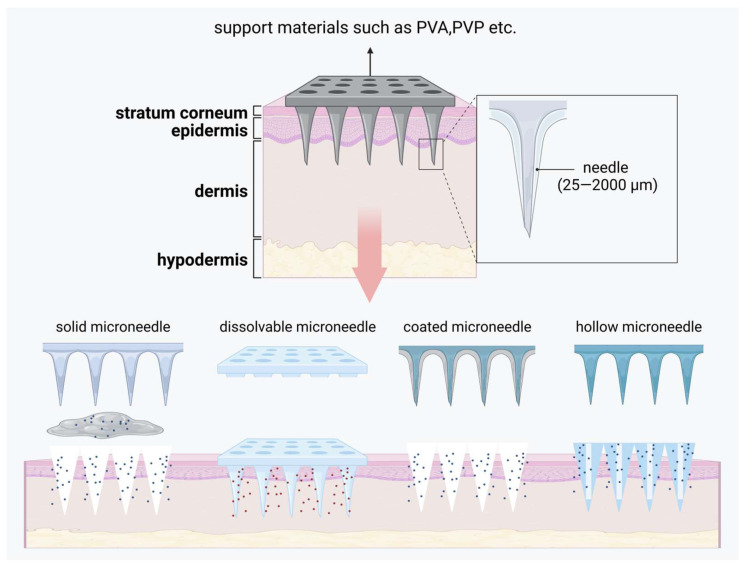
A brief introduction to microneedle and how it is released. Created with BioRender.com.

Microneedle (MN) technology is typically used to enhance transdermal drug delivery into systemic circulation. Made from biocompatible materials, these needles range in length from several hundred micrometers to a few millimeters, allowing them to penetrate the stratum corneum without reaching the nerve and blood vessels in the dermis, thereby minimizing pain and bleeding [147]. Various types of microneedles have been developed, including solid microneedles, dissolvable microneedles, coated microneedles, and hollow microneedles [148]. A microneedle system primarily consists of two components: invasive needles and a support base. The invasive part is composed of numerous needles, each measuring approximately 25 to 2000 μm in length, while the support base ensures that the needle tips can effectively penetrate the stratum corneum [149]. A brief introduction to microneedling is given in Figure 5.

In psoriasis treatment, microneedle technology offers precise dosage control and stable drug release, significantly enhancing transdermal drug absorption, minimizing systemic side effects, and simplifying the procedure, making it more suitable for patient self-administration [150]. Dai [151] et al. developed a calcipotriol monohydrate (CPM)-loaded microneedle using nanotechnology to enhance skin penetration and prolong skin retention. The invasive component of this microneedle consists of CPM in a nanosuspension form combined with polyvinylpyrrolidone (PVP) and polyvinyl alcohol (PVA) with a 3D-printed polylactic acid (PLA) backing. Compared to traditional creams, this microneedle exhibits superior mechanical strength, allowing effective skin insertion, and its rapidly dissolving needle tips enhance drug delivery efficiency. Bi [152] et al. formulated a novel microneedle system comprising MTX/EGCG-HP gel as the invasive material and PVA/PVP as the support matrix. Once inserted into the skin, this microneedle extends drug retention time, enables rapid diffusion, and offers ROS-responsive release, optimizing therapeutic outcomes. Wang [153] et al. developed a hyaluronic acid-based microneedle to enhance the delivery of β-elemene, investigating, for the first time, its direct pro-apoptotic effects on psoriatic keratinocytes. This approach also suppresses inflammation in psoriatic keratinocytes by downregulating IL-1, IL-6, and IL-8 expression.

### 5.3. Dressing-Based Transdermal Delivery for Psoriasis Treatment

**Figure 6:**
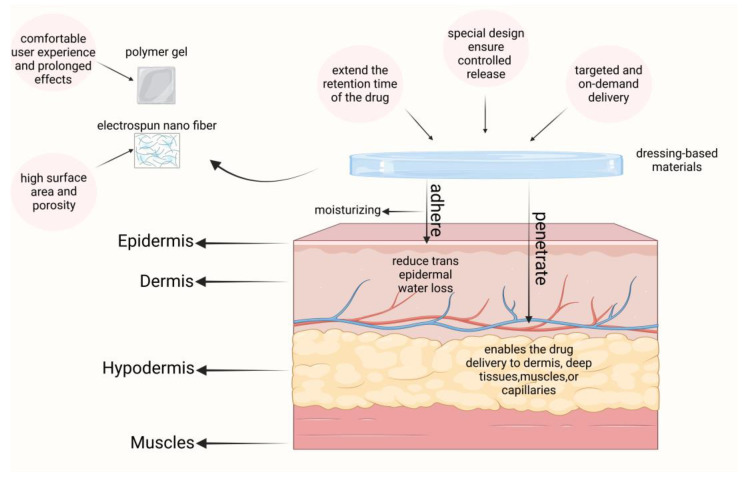
A brief introduction to dressing-based carriers and their role in psoriasis. Created with BioRender.com.

Dressing-based transdermal delivery systems can extend the retention time of drugs on the skin, thereby enhancing the therapeutic efficacy of topical medications. Additionally, these systems help to limit transepidermal water loss (TEWL) and may even provide hydration to the skin, facilitating the transport of drugs into the dermis, deeper tissues, muscles, or capillaries. By adjusting the properties of the polymers used in these dressings, it is possible to control drug release and achieve targeted, on-demand delivery. We briefly schematize this class of materials in Figure 6. Such systems are increasingly applied in psoriasis treatment, with notable examples including polymeric gels and electrospun nanofiber materials.

#### 5.3.1. Polymeric Gels for Psoriasis Treatment

Gel formulations offer significant advantages in transdermal delivery systems, primarily due to their soft texture, breathability, and high viscoelasticity. These properties not only provide a comfortable user experience but also enable controlled drug release for prolonged therapeutic effects. Common materials used in gel formulations include carbomers, cellulose derivatives, propylene glycol, and glycerin. These materials combine with solvents like water or glycerin to form a uniform, smooth gel matrix that supports stable drug release. Additionally, gel formulations allow for customization based on the specific characteristics and therapeutic needs of the drug, enabling personalized treatment. For instance, incorporating environmentally responsive materials can create smart gels sensitive to temperature, pH, or other factors, further optimizing drug delivery and therapeutic efficacy.

Kaempferol, a flavonoid found in vegetables, fruits, and spices, is renowned for its antioxidant and anti-inflammatory properties. However, its poor water solubility and bioavailability limit its clinical application. Su [154] et al. developed a hydrogel (DK-pGEL) using Pluronic F127 combined with a deep eutectic solvent (DES) containing varying concentrations of kaempferol, which inhibited the proliferation of HaCaT cells without significant cytotoxicity, exhibited antioxidant effects, reduced ROS production, and provided hydration to damaged skin, making it a promising candidate for clinical treatment. Darshan R. Telange’s team [155] reported an eco-friendly silver nanoparticle (AgNP) gel formulation using Pongamia pinnata seed extract to load AgNPs. The gel matrix, based on carbopol 980P and Cremophor A25, enhanced the retention, accumulation, and penetration of AgNPs into psoriatic skin. The AgNPs were formed through a mechanism involving recognition, reduction, and nucleation, with the optimized formulation showing improved retention and accumulation in psoriatic lesions, offering a potential therapeutic option for psoriasis.

#### 5.3.2. Electrospun Nanofiber Materials

Electrospinning was a versatile and straightforward technique for producing ultrafine fibers by drawing charged liquid jets into fibers with diameters ranging from micrometers to tens of nanometers [156]. In drug delivery systems, electrospinning could create nanofiber carriers capable of effectively loading and releasing anti-psoriatic drugs, such as MTX. The high surface area and porosity of electrospun nanofibers would improve drug bioavailability and penetration, which is especially beneficial in psoriasis.

Hydrocortisone (HQ) remains a first-line treatment for psoriasis; however, due to issues related to formulation aesthetics and patient adherence, there is an urgent need for new drug delivery formats. Abbas Hemati Azandaryani’s team [157] developed a nano bandage using electrospinning technology, incorporating HQ with polyacrylonitrile (PAN) under conditions of 0.75 mL/h flow rate and 14 kV voltage. In vitro drug release reached approximately 40%, with increased release rates as surfactant levels rose. Tests on HUVEC cell lines indicated no cytotoxicity, and the nano bandage exhibited high mechanical strength, making it a promising alternative for psoriasis treatment.

## 6. Recent Patents and Clinical Trials

Currently, and for a long time to come, the treatment of psoriasis will continue to be based on topical treatments, small-molecule oral agents, and biologics. Since Bristol–Myers Squibb released deucravacitinib, the world’s first oral TYK2 inhibitor for psoriasis, many ‘big pharms’ have taken notice of a target that was once thought to be ‘un-druggable’! The target was once thought to be ‘undruggable’. Based on Phase 2b results, such as Takeda-guided TAK-279, which showed a strong overall clinical benefit and, importantly, had a significant number of patients achieve near-complete or complete skin clearance with a PASI of 90 or 100, Takeda will initiate a Phase 3 study of TAK-279 in psoriasis in the fiscal year 2023 (https://www.takeda.com/newsroom/newsreleases/2023/takeda-announces-positive-results-in-phase-2b-study-of-investigational-tak-279/, accessed on 28 December 2024). In addition to Takeda, there are also Pfizer and BeiGene who are also following up on this target. We are confident that even better and less expensive psoriasis treatment drugs will emerge in the near future, bringing good news to patients. We have collated recent clinical trials and patents for drugs used to treat psoriasis in Table 4.

Nanoformulations offer significant advantages, including improved drug stability, prolonged in vivo circulation, and targeted delivery, making them highly promising for enhancing tissue distribution and increasing drug bioavailability. Compared to conventional formulations, novel drug delivery systems have limited market availability. This is likely due to three key challenges: high production costs, stability issues, and limited accessibility. We believe that nanotechnology will play an important role in the future treatment of psoriasis. To protect the rights of innovators, it is essential to safeguard research that yields significant results. Table 5 summarizes some of the recent psoriasis-related patents.

## 7. Future Perspectives and Conclusions

We have reviewed the pathogenesis, clinical diagnostic methods, and commonly used clinical treatments for psoriasis, with a detailed discussion on the challenges and progress of novel transdermal drug delivery systems in psoriasis therapy. Psoriasis is becoming an increasingly serious clinical issue worldwide. Although its pathogenesis is complex, ongoing research is gradually unraveling its mysteries. Currently, psoriasis diagnosis primarily relies on physical examination by physicians, supplemented by instrumental diagnostics. In the future, by investigating the genetic background and immune response abnormalities associated with psoriasis, we may identify reliable biomarkers that enable earlier and more accurate diagnosis. Recent studies on cellular signaling pathways have provided promising new directions for psoriasis treatment, such as the development of biologics and drugs targeting inflammatory pathway factors. These therapies offer rapid results but often come with high costs. For example, the biologic drug secukinumab (Cosentyx) is priced at CNY 3000 for two doses on Chinese online platforms. Given the chronic and lifelong nature of psoriasis and the need for ongoing treatment, this can impose a significant financial burden on families.

Currently, medications for psoriasis are primarily administered orally, by injection, or topically. Oral medications often cause gastrointestinal side effects and have low bioavailability. Injectable drugs, while effective, face issues with patient compliance, systemic toxicity, and high production costs with stringent quality requirements. Topical medications, on the other hand, can greatly improve patient compliance, maintain stable drug levels in the blood, and reduce side effects, making them an important approach in psoriasis treatment. However, psoriasis is characterized by significant structural and functional changes in the skin barrier, leading to excessive epidermal proliferation and keratinization. Conventional transdermal delivery systems, such as ointments and creams, often fail to sufficiently deliver and retain the medication at the target site (i.e., the deeper epidermis and dermis) or may cause systemic side effects due to the drug entering the bloodstream.

The development of new transdermal drug delivery systems offers a unique opportunity for creating effective and low-toxicity treatments. For instance, a nanotechnology-based transdermal delivery system with a small size allowed deeper penetration into the skin layers while also providing benefits such as reduced dosage, increased dose dependency, higher encapsulation efficiency, controlled release, enhanced surface area, and improved bioavailability; a microneedle-based transdermal delivery system could penetrate psoriatic lesions to deliver drugs with minimal damage. However, research on novel transdermal drug delivery systems for psoriasis is still in its early stages, and there are numerous challenges, from laboratory research to market launch. The future clinical translation of these systems depends not only on their potential efficacy but also on advancements in large-scale production. Therefore, there is an urgent need not only for the exploration of new carrier systems or combination therapies but also for a focus on scalability to enable earlier market entry and deliver greater societal benefit.

## Figures and Tables

**Table 1:** Auxiliary diagnostic methods for psoriasis.

Diagnostic Methods	Inspection Items	Diagnostic Criteria	Precautions	Ref.
Blood Routine Test	White blood cell count, erythrocyte sedimentation rate, and platelet count in blood	Elevated white blood cell count, increased erythrocyte sedimentation rate, and elevated platelet count, among other things	Maintain an empty stomach to avoid compromising diagnostic results	[56]
Histopathology	Skin tissue samples for microscopic examination	Thickening of the stratum corneum, hyperkeratosis, hypertrophy of the stratum spinosum, and vasodilatation of the papillary layer of the dermis	Ensure representative sampling of skin tissue from lesions and take care to avoid topical medications from affecting results	[57]
Dermoscopy	Patient’s area of skin damage	Bright red background, regularly or circumferentially distributed punctate blood vessels, and diffusely distributed white scales	Dermoscopic test results should only be used as a reference, and pathologic biopsies are also performed when necessary to prevent misdiagnosis	[58]
Biochemical Test	Liver function, kidney function, and blood lipids	Abnormal liver function, hypertriglyceridemia, low HDL, etc.	Maintain a fasting state to avoid the influence of food on the test results	[59]
Immunological Test	Immunoglobulins, complement and cytokines, etc.	Elevated immunoglobulins, decreased complement, and abnormal cytokine levels	Maintain a fasting state to avoid the influence of food on the test results	[60]
Digital X-ray photography	Patients with symptoms such as joint pain or swelling	Evaluate joints for lesions such as narrowing of the joint space, osteoporosis, osteophytes, or other changes in joint structure	Some radiation to the human body, metal inserts in the body need to be notified	[61]
Computed Tomography (CT)	Patients with suspected or diagnosed arthropathic psoriasis	Signs of bone erosion, osteophytes, and narrowing of the joint space at the margins and center of the joints	Remove all metal objects from the body; inform the body of the presence of metal implants; pregnant women should minimize CT examinations to avoid affecting the development of the fetus	[62]
Magnetic Resonance Imaging (MRI)	Patients with suspected or diagnosed arthropathic psoriasis	Ancillary X-rays to assess the extent of inflammation, joint cavity effusion, synovial thickening, bone marrow edema, inflammation of tendons and ligaments, and bone destruction	MRI is poorly reported in the literature, and there is no consensus on the criteria for examining to evaluate activity and bone structure changes in psoriatic arthritis	[63]

**Table 2:** Current types of drugs used in the clinical treatment of psoriasis, advantages, and disadvantages summarized.

Treatment	Types of Drugs	Used Drugs	Advantages	Disadvantages	Ref.
Oral medicines	Immunosuppressants	Methotrexate	Convenient, easy to store, relatively inexpensive	liver and kidney damage	[66]
		Apremilast		Short-term gastrointestinal side effects	[67]
	Vitamins derivatives	Acitretin		Elevated serum triglycerides and, to a lesser extent, elevated serum cholesterol and liver transaminase levels	[68]
	Glucocorticosteroid	Methylprednisolone		Hyperglycemia, hypertension, osteoporosis, immunosuppression, weight gain, and bradycardia and cardiac arrhythmias	[69]
		Prednisone		Possible skin atrophy	[70]
		Dexamethasone		Adrenal function effects	[71]
	TCM	Xiaoyin jiedu granules		Less researched, unknown side effects	[72]
Injectable medications	Immunosuppressant	Methotrexate	Effective, lower dosing frequency, more economical	Injection site edema, pimples, erythema, etc.	[73]
		Hydroxyurea		Hyperpigmentation, dryness, and skin atrophy	[74]
		Azathioprine		Pemphigus erythematosus rash and fever	[75]
	Biological agents: TNF-α inhibitor	Etanercept	Outstanding therapeutic effect, good specificity	Allergic reactions, immunodeficiencies, sepsis, tuberculosis (reactivated or novel infections), and rare lymphoma risks	[76]
		Infliximab		Itching, erythema, rash, and other rare side effects such as induced psoriasis in Crohn’s patients	[77,78]
		Adalimumab		Injection site reactions, rare side effects include congestive heart failure, lupus-like syndrome, lymphoma promotion, neurological disorders	[79]
		Certolizumab Pegol		Upper respiratory tract infections, skin rashes, and urinary tract infections	[80]
	Biological agents: IL-23 inhibitor	Guselkumab		Infections, nasopharyngitis, etc.	[81]
		Tildrakizumab		Nasopharyngitis, headaches, upper respiratory infections, and worsening psoriasis	[82]
	Biological agents: IL-23 inhibitor	Risankizumab		Pneumonia, cerebrovascular accidents, cataracts, loss of consciousness, heart disease, cirrhosis of the liver and thrombosis	[83]
	Biological agents: IL-17A inhibitor	Secukinumab		Nasopharyngitis, diarrhea, upper respiratory infections, itching and headaches	[84]
		Ixekizumab		Nasopharyngitis and injection site reactions	[85]
		Brodalumab		Suicidal ideation and behavior, Nasopharyngitis, headaches, upper respiratory infections and joint pains	[86,87]
	Biological agents: IL-36 receptor inhibitor	Spesolimab		Drug reactions such as eosinophilia and systemic symptoms (DRESS), cholelithiasis, and breast cancer	[88]
	Biological agents: IL-12/23 inhibitor	Ustekinumab		Inhalation tract infections, nasopharyngitis, headache and injection site reactions, and a small number of serious infections or cardiovascular adverse events	[89]
	Biological agents: IL-17A/F inhibitor	Bimekizumab		Nasopharyngitis, upper respiratory tract infections, oral candidiasis, headaches and diarrhea	[90]
Topical medicines	Vitamin derivatives	Calcipotriol	Lower side effects than systemic administration, protects the skin barrier, less expensive	Possible transient hypercalcemia, prolonged use of calcipotriol may cause local skin reactions, which may lead to non-compliance; approximately 20% of patients do not respond to calcipotriol treatment	[91]
	Tar preparations	Coal tar detergent		Allergic dermatitis, folliculitis, occupational acne, epidermal atrophy and hyperpigmentation	[92]
	Glucocorticosteroid	Fluticasone propionate, Mometasone furoate		Itching, burning, dryness, stinging, folliculitis, irritation or worsening of skin conditions Possible diabetes, skin atrophy	[93,94,95]
	Calcineurin inhibitors	Tacrolimus		Poor penetration appears to be relatively ineffective in the treatment of plaque psoriasis	[96]

**Table 3:** Recent work in the development of nanotechnology-based transdermal delivery for psoriasis treatment.

Delivery Carrier	Materials	Drug	Size and Zeta Potential	Mechanism and Efficacy	Ref.
Metal nanocarriers	Gold nanoparticles functionalized with 3-mercapto-1-propansulfonate	Methotrexate	5 nm, −32 ± 1 mV	Reduction of induced keratinocyte hyperproliferation, epidermal thickness, and inflammatory infiltrate in a psoriasis-like mouse model	[118]
	Spherical nucleic acid nanoparticle conjugates	-	12.38 ± 1.59 nm, −28 mV	Stable and non-toxic. Significantly reduces gene expression in cells, and in vivo experiments show that S significantly inhibits cell proliferation	[119]
Polymer nanocarriers	Diblock copolymer of polyethylene glycol and polypropylene sulfide	Deucravacitinib	105 nm	Longer retention time in the dermis inhibits the STAT3 signaling cascade, with corresponding reductions in the levels of the differentiation and proliferation markers Keratin 17 and Cyclin D1, respectively	[120]
Polymer nanocarrier	Gelatin-oleic acid coupling modified with 4-(3-boronophenylamino)-4-oxobutanoic acid	Celastrol	200–300 nm, −6.00 ± 0.10 mV	Improved cellular and skin penetration and enhanced antipsoriasis activity in a mouse model	[121]
	Methoxypolyethyleneglycol-thioether-thiols	Calcipotriol	283 ± 13.1 nm, −14.9 mV	ROS sensitivity, good biocompatibility, safe routes of administration, short treatment cycles, etc.	[122]
	Solvent-free ion-gelation of chitosan	Tacrolimus	140.8 ± 50.0 nm, 17–33 mV	Successful, repeatable, and simple. It eliminates the use of any harmful organic solvents and is superior to Talmos^®^ Ointment in its efficacy in the treatment of plaque psoriasis	[123]
	Lecithin–chitosan hybrid	Tacrolimus	160.9 ± 15.9 nm/118.7 ± 13.3 nm, 11–42 mV	Successfully doped with hydrophobic drugs; the addition of suitable co-solvents maximized the drug-carrying capacity of the above hybrid particles for hydrophobic drugs, and the efficiency of the treatment of psoriasis was superior to that of commercially available tacrolimus ointment	[124]
	Hyaluronic acid-modified chitosan	Gallic acid	220.1 ± 0.18 nm, −2.104 ± 0.34 mV	Reduces epidermal hyperproliferation and associated inflammation and exhibits negligible systemic toxicity	[125]
	Kolliphor^®^ 407 Coupling	Mycophenolic acid	Around 20 nm	Higher critical micelle concentration. Improved antiproliferative effect on TNF alpha-induced HaCaT cell proliferation	[126]
Polymer nanocarrier	Polaxomer F127 and P123 designed by the Quality by Design (QbD) methodology.	Resveratrol	142.67 ± 6.98 nm, −35.65 mV	Better skin penetration and enhanced resveratrol deposition in deeper skin layers enhanced the expected therapeutic effect of topical treatment of plaque-like psoriasis-like skin disease	[127]
	Poly(2-(dimethylamino)ethyl methacrylate) (PDMA) grafted hairy silica particles (cSPs) with tunable PDMA length and particle size	-	700 nm, +53.1 mV	enhance drug accumulation and prolong retention time in psoriatic lesions, leading to excellent treatment results. Ability to target cell free DNA	[128]
	Capryol 90/MCT oil, glycerin, Absolute ethanol	Alpinia galanga extract	60.81 ± 18.88 nm, −7.99 ± 4.14 mV	Useful for restoring radiant skin texture and effectively treating psoriasis in a mouse model	[129]
	Propylene glycol dicaprylocaprate, Diethylene glycol monoethyl ether, Polyoxyethylene sorbitan monolaurate	Curcumin	10.57 nm, −18.7 mV	It is possible to improve the topical efficacy of poorly penetrating Curcumin for the long-term management of psoriasis	[130]
Lipid nanocarriers	Peptide TD-coupled liposomes	Curcumin	Around 100 nm	Increased transdermal efficiency of curcumin	[131]
	Mannosylated liposomes	Celastrol	85.5 ± 0.7 nm, −6.0 ± 0.3 mV	Enhancement of DC uptake and induction of DC tolerance	[132]
	Transfersomes	Methotrexate and Baicalin	150.20 ± 3.57 nm, +38.6 mV	Better permeability	[133]
	DOTAP liposome	Bexarotene	67.8.2 ± 7.15 nm, 46.4 ± 2.8 mV	Increased drug penetration and effective reversal of psoriasis	[134]
	Ceramide/Phospholipid Composite Cerosomes	Cyclosporine and Dithranol	222.36 ± 0.36 nm, 29.36 ± 0.38 mV	Increased skin permeability and anti-proliferation	[135]
	Lipoid S 100, ethanol, and Phosphatidylcholine	Psoralen	120.77 ± 22.43 nm	Good biocompatibility, low cytotoxicity	[136]

**Table 4:** Recent clinical trials of drugs used to treat psoriasis. Data obtained from https://clinicaltrials.gov/, accessed on 28 December 2024.

Type	Drug Name	Controlled Substance	Research Target	Clinical Trials Status	Country or Region	Item No.
Topical therapies	SGX302 (topical hypericin ointment and gel)	placebo	Patients with Mild to Moderate Psoriasis	Phase 2 Study Evaluating	USA	NCT05442190
	Sericin extract and turmeric extract cream	Triamcinolone acetonide 1% Cream	Patients over 18 years of age with psoriasis	/	Thailand	NCT06482398
	Tapinarof cream	/	Plaque Psoriasis in Pediatric Subjects	Phase 3	USA, Canada	NCT05172726
	Chitosan Nanocrystalline Qinteng Huoxue Runji Ointment	placebo	Psoriasis with Blood Stasis Syndrome	/	China	NCT06396013
	ZL-1102 Topical gel		Chronic Plaque Psoriasis	Phase 2	Australia	NCT06380907
Oral medicines	GM-XANTHO	placebo	Mild to Moderate Psoriasis	Phase IIa clinical trial	/	NCT06620692
	TAK-279	/	Generalized Pustular Psoriasis or Erythrodermic Psoriasis	Phase 3	Japan	NCT06323356
	Piclidenoson	Placebo	Moderate-to-Severe Plaque Psoriasis	Phase 3	/	NCT06643260
	Shuiniujiao Dihuang Decoction	Placebo	Mild to moderate plaque Psoriasis		Hong Kong	NCT05815797
	LY4100511	Placebo	Moderate-to-severe plaque Psoriasis	Phase 2	USA	NCT06602219
	Jiuweihuaban Pill	Placebo	Moderate to Severe Plaque Psoriasis (Syndrome of Blood-heat)	Phase II	China	NCT06058546
Oral medicines	ESK-001	Apremilast, placebo	Moderate to Severe Plaque Psoriasis	/	USA, Canada	NCT06586112
Injectable medications	Enoxaparin	/	Plaque Psoriasis	/	/	NCT06416566
	Tildrakizumab (biological agent)	/	Psoriasis in specific areas such as scalp, nails, genital area, and palmoplantar localization	/	France	NCT05938361
	HB0017 (biological agent)	placebo	Moderate to Severe Plaque Psoriasis in adults	phase III clinical study	China	NCT06477237
	CMAB015 (biological agent)	Secukinumab	Moderate to Severe Plaque Psoriasis in adults	/	China	NCT06398652
	Tildrakizumab (biological agent)	/	Genital Psoriasis	/	Austria	NCT06029257
	Netakimab (biological agent)	Placebo, Adalimumab	Children with Moderate to Severe Plaque Psoriasis	/	Russia	NCT06640517
	Botulinum Toxin-A	/	Psoriasis Vulgaris	/	/	NCT06203470

**Table 5:** Recent patents of drugs used to treat psoriasis. Data obtained from https://www.epo.org/en, https://patents.google.com/, accessed on 28 December 2024.

Type	Drug	Use	Country or Region	No.
/	New peptide from the alpha1 adrenergic receptor	Peptides (I) from the alpha 1-adrenergic receptor (AR), or their mutants or variants, form an epitope that can bind to autoantibodies (AAb) present in patients with psoriasis	German	DE10041560A1
/	CDK4/6 inhibitor	Inhibitor for the suppression of the cellular IκBζ expression	German	EP3797776A1; US2022280511A1; WO2021063734A1
Oral medicines	Dimethyl fumarate	Psoriasis treatment	Spanish	WO2020053218A1
Topical therapies	FLUVOXAMINE	Psoriasis treatment	Portugal	EP4469032A1; PT117765A; WO2023146426A1
/	SIALIC ACID	Psoriasis treatment	Norway	CN114450026A; EP4003365A1; US2022288095A1; WO2021019295A1
/	CGRP receptor antagonists and/or pharmaceutical compositions	Methods are useful for treating, ameliorating, alleviating, providing for prophylaxis or prevention of, halting the progression of, and/or reducing the risk of psoriasis in a mammalian subject, such as a human	Ireland	AR126954A1; WO2023034466A1
Injectable medications	IL-17 antagonists (biological agent)	Modifying the psoriasis disease course in particular patients having chronic plaque-type psoriasis and inhibiting the progression to PsA in these patients, as well as medicaments, dosing regimens, pharmaceutical formulations, dosage forms, and kits for use in the disclosed uses and methods.	Switzerland	WO2018158741A1
/	Oligonucleotide complementary to the sequence of human miR-203b-3p microRNA	Psoriasis-induced itching	Italy	EP4437105A1
Topical therapies	Composition comprising calcipotriol and betamethasone as active ingredients, petrolatum, and a propellant	Use in maintenance treatment of patients with psoriasis who are in remission.	Denmark	EP4041186A1
Oral medicines	Small molecule inhibitors of tumor necrosis factor-alpha	Psoriasis treatment	France	WO2024223740A1
Topical therapies	Medicament formed of a hydrogel and coal tar and/or coal tar extract is held	The dressings may be adapted for use in conjunction with phototherapy from UV light sources	USA	US10058711B2
/	Comprising PPAR agonists and Nrf2 activators and methods of using combinations of PPAR agonists and Nrf2 activators	Psoriasis treatment	Australia	AU2021201390B2
Topical therapies	An active agent comprising at least one steroid in the form of topical sprays that are propellant-free, and/or substantially non-foaming, and/or alcohol-free	Psoriasis treatment	USA	US20210386758A1
/	Anti-interleukin antibodies	Psoriasis treatment	Japan	JP7558326B2
/	Anti-IL-12 antibody	Production of antibodies or portions for treating and/or diagnosing IL-12-related conditions, diseases, and disorders	USA	US9409984B2
Topical therapies	Psoriasis nanoemulsion tincture	Ppina gleditsiae and radix platycodonis nanoemulsion tincture; radix et rhizoma cynanchi paniculati and cortex dictamni meridian freeing, blood activating, heat-clearing, dampness drying, parasite removing, wind dispelling, and itching arresting relieving nanoemulsion tincture	China	CN106668689A
Topical therapies	Tripterine analog pristimerin-loaded exosome composite nanohydrogels for targeted therapy	The hydrogel delivers the drug to the psoriasis site in a targeted manner through PD-1/PD-L1 specificity to play a role in psoriasis, thus realizing targeted treatment of psoriasis, improving the microenvironment of the disease, lowering the toxicity and side effects of the drug, and improving the therapeutic effect	China	CN116059158A
Topical therapies	Long-acting gel preparation containing arsenic trioxide nano-liposome	After transdermal administration, arsenic trioxide can slowly penetrate and accumulate in the diseased area at a stable concentration. The long-acting gel preparation prepared by the present invention promotes efficient transdermal absorption of the drug, increases the retention of arsenic trioxide in the skin, improves the local concentration of the drug, and enhances the therapeutic effect on psoriasis	China	CN116270425A
Topical therapies	TCM micro-nano fiber film	Topical Chinese medicine micro-nano-fiber film for psoriasis treatment has the advantages of low cost, convenience, direct action on the affected area, fast onset of action, short course of treatment, not easy to recur, no toxic side effects and so on	China	CN114960028A

## Data Availability

Not applicable.
